# Late‐onset PTSD and coping strategies for frontline nurses during the COVID‐19 epidemic in China

**DOI:** 10.1002/nop2.1018

**Published:** 2021-08-15

**Authors:** Yongfang Jiang, Bowen Hu, Baoren Tu, Quan Zhuang

**Affiliations:** ^1^ Transplantation Center The 3rd Xiangya Hospital Central South University Changsha China; ^2^ Department of Infectious Diseases The 2nd Xiangya Hospital Central South University Changsha China; ^3^ Xiangya School of Medicine Central South University Changsha China; ^4^ Research Center on Transplantation Medicine of National Health Ministry Changsha China

**Keywords:** coping strategy, COVID‐19, frontline nurse, late‐onset PTSD, social support

## Abstract

**Aim:**

To evaluate the late‐onset post‐traumatic stress disorder (PTSD) situation, coping strategies and social supports for frontline clinical nurses 6 months after involvement in fighting against coronavirus disease 2019 (COVID–19) outbreak in China.

**Design:**

A cross‐sectional design.

**Methods:**

We recently randomly distributed a questionnaire online to Chinese nurses who had taken care of COVID‐19 patients since the end of January 2020. The questionnaire was made up of three professional scales, the impact of event scale‐revised, simplified coping style questionnaire and social support rating scale included.

**Results:**

The general prevalence of late‐onset PTSD among frontline nurses was 88.19%. Nurses who worked in Hubei Province (the kindle place of the COVID‐19 outbreak) showed lower risk of late‐onset PTSD symptoms than those who did not. We also found that positive coping strategies were correlated with less late‐onset PTSD symptoms. Meanwhile, getting more social supports could help these nurses to obtain positive coping strategies.

## 1 INTRODUCTION

The coronavirus disease 2019 (COVID‐19) pandemic has been initially reported in Hubei Province, China (Zhu et al., 2020), and has been seriously endangering human health and life worldwide. As of 6 November 2020, over 48.5 million COVID‐19 cases and 1.2 million deaths have been reported (World Health Organization, 2020). Because of the widespread and serious spread of the disease, a large number of medical workers are needed in the fight against the epidemic, and nurses are the largest group among them. Chinese frontline clinical nurses had begun the fight against the COVID‐19 epidemic since the end of January 2020. Under this circumstance, during this catastrophic epidemic, they were not only under great physical challenges but also faced high psychological stress. Excessive workload has been verified as a statistically significant stressor for nurses, which would affect their mental health (Maharaj et al., 2018; Watanabe & Yamauchi, 2018). Several studies have investigated that medical staff experienced psychological conditions, such as anxiety and depression (Chen et al., 2020; Lai et al., 2020). Post‐traumatic stress disorder (PTSD) is a mental disorder defined by intrusive recollections, avoidant behaviour and hyperarousal following the experience of a horrifying traumatic event (Kirkpatrick & Heller, 2014). PTSD symptoms often come out 1–6 months after the triggering event. Late‐onset PTSD, nonetheless, may be easily overlooked, diagnosed over 6 months after the traumatic event or termination of long‐term exposure (Utzon‐Frank et al., 2014). At present, this COVID‐19 epidemic in China is largely under control, and the post‐epidemic era is coming. However, the influence of this late‐onset psychological stress is gradually coming out.

Coping strategies are vital to influence individual stress levels (Labrague et al., 2017). Coping strategies usually consist of positive and negative categories. When faced with stress, individuals with positive coping strategies have positive thoughts and solutions (e.g. constructive actions). In contrast, negative coping strategies are recognized as palliative coping strategies and negative appraisals under stressful conditions (e.g. avoidance) (Ding et al., 2015). Moreover, several studies suggested that nurses adopting positive coping strategies had a statistically significant correlation with their better job performance and satisfaction, which can promote patients' safety (Li et al., 2017; Zhou et al., 2017). Therefore, positive coping strategies of nurses against the COVID‐19 pandemic would be a crucial factor in nurses' mental health and patients' outcome.

Social support is considered another important factor influencing mental health (Wang et al., 2017). Social support comes from one's social environment, including family members, friends, colleagues, parties, communities, media, unions and even society. Previous research identified that good social support could positively affect mental health (Saltzman et al., 2020). In addition, social support may affect coping strategies (Tesfaye, 2018). Consequently, the late‐onset PTSD situation of frontline clinical nurses may be influenced by their social supports.

From the above, coping style and social support may be closely related to late‐onset PTSD. Several studies are trying to figure out the relations between them. Such as a study conducted to define social support and coping as predictors of PTSD among traffic survivors (Okech et al., 2018). Another study focused on the influence of social support and coping style on chronic PTSD after floods and found that better social support, the positive coping style could significantly improve the long‐term prognosis of patients with PTSD (Dai et al., 2016).

Therefore, our study is to assess the late‐onset PTSD, coping strategies and social supports situation for frontline clinical nurses 6 months after involvement in fighting against the COVID‐19 outbreak in China, call for more attention to the mental health problems of these nurses and give timely and sufficient support to them.

## 2 METHODS

### 2.1 Study overview

A questionnaire was distributed online to Chinese nurses who had directly contacted COVID‐19 patients since the end of January 2020. The questionnaire was made up of three professional scales, IES‐R, SCSQ and SSRS included. Then, the late‐onset PTSD, coping strategies, social supports and their associations for frontline nurses were assessed. Several steps are taken when conducting this study and involve:

- Finding the aim of our study, which is the late‐onset PTSD situation, coping strategies for frontline clinical nurses after COVID‐19 outbreak in China;
- Defining the study setting and inclusion criteria of the patients;
- Designing a questionnaire based on 3 scales (impact of event scale‐revised, simplified coping style questionnaire and social support rating scale)
- Distributing the questionnaires and collecting;
- Assessing late‐onset PTSD situation by IES‐R score, assessing coping strategies according to SCSQ scale and evaluating social support by SSRS checklist;
- Data analysis, participants were divided into “Hubei” and “non‐Hubei” groups and student *t* test was used to compare the differences between them; in addition, Pearson's correlation and regression analysis were used, which is explained in more detail below,

### 2.2 Study setting and participants

We conducted a cross‐sectional study at the end of July. Firstly, we designed the electrical questionnaire by Questionnaire Star, a professional online survey platform in China. Then, the questionnaires were randomly distributed to frontline nurses fighting against the COVID‐19 epidemic by Wechat, which is China's largest real‐name communication app by a number of users. Moreover in this way, we can make sure all the people who filled out the questionnaire were frontline nurses. 873 questionnaires were received from 28th July to 30th July, of which 9 were ruled out because they were finished in 2 min, and the answers were highly consistent. Therefore, a total of 864 questionnaires were qualified finally. Hubei Province is believed to be the first place where COVID‐19 became popular in China and where the epidemic is much more severe than in other places in China. We divided these frontline nurses into 2 groups. One group was those who had worked in Hubei Province (the kindle place of COVID‐19 outbreak) during the COVID‐19 epidemic, and the other group was the ones that had worked outside Hubei Province. All participants provided written informed consent, and participants were anonymous. It is not required by our institution to obtain research ethical committee approval for a survey with a non‐clinical sample and anonymized data, but we still obtained approval for the study protocol from the institutional review board (Ethics Committee) of the 3rd Xiangya Hospital, Central South University (20005‐IRB).

### 2.3 Measurement of PTSD

Post‐traumatic stress disorder was assessed by the impact of event scale‐revised (IES‐R) checklist (Noda et al., 2018). It can be used to evaluate the performance and severity of PTSD, and it is also a good predictor for the occurrence of PTSD. There are 22 items in this scale, including three dimensions of invasive symptoms (8 items), avoidance symptoms (8 items) and high arousal symptoms (6 items), and each item with a Likert rating scale from 0–4. The Cronbach's alpha was 0.89, the split‐half reliability was 0.93 and the relevance validity of the scale was 0.55. The score interval of the IES‐R scale was as follows: 0–8 were divided into subclinical symptoms; 9–25 are classified as mild; 26–43 are classified as moderate; and 44–88 are classified as severe.

### 2.4 Measurement of coping strategies

The coping strategies were measured by the simplified coping style questionnaire (SCSQ) (Su et al., 2018). The SCSQ is a 20‐item instrument consisting of 2 subscales: positive coping (12 items) and negative coping (8 items). Each item of the SCSQ ranked on a 4‐point Likert scale ranging from 0 points to 3 points. The SCSQ had adequate content validity, internal consistency and test–retest reliability in Chinese (Xiao, 1994). In this study, the Cronbach's alpha of the positive coping and negative coping were 0.904 and 0.877, respectively.

### 2.5 Measurement of social support

Social support was measured by the social support rating scale (SSRS) (Xiao et al., 2020). The SSRS is a 10‐item instrument consisting of 3 subscales: objective social support (3 items), subjective social support (4 items) and support utilization (3 items). Items 6 and 7 were rated on a 9‐point Likert scale ranging from 0 points to 8 points; the other items are recorded on a 4‐point Likert scale ranging from 1 point to 4 points. Higher scores indicate higher levels of social support. In the present study, the Cronbach's alpha of the 3 subscales ranged from 0.678–0.756.

### 2.6 Data analysis

Questionnaire results were summarized from the imported Excel file and analysed using SPSS version 18.0 software (IBM Corporation). Descriptive statistics were used to present the sample characteristics and study variables. Continuous variables were expressed as an average with a standard deviation (*SD*). Categories variables were expressed as numbers and percentages. Student *t* test was used to compare the differences in IES‐R, SCSQ and SSRS scores between “in Hubei” and “outside Hubei” groups. Student *t* test was used to compare the differences in IES‐R scores between “male” and “female” groups. The level of significance was set as 0.05. Pearson's correlation and regression analysis were used to analyse the relationship between social support, coping strategies and late‐onset PTSD symptoms. Analysis of variance and coefficient of determination (R square) were calculated to test the linear model. A Q‐Q Plot was made by SPSS to check the normality, linearity and heteroscedasticity of the residuals. Thus, the rationality of regression analysis was verified.

## 3 RESULTS

### 3.1 Participant characteristics

A total of 864 participants completed this questionnaire. 77.78% of the participants were aged between 25–39 years old, which is the main population of the nursing staff. Most participants were female (92.01%). Of the 864 participants, 211 (24.42%) had worked in Hubei Province during this epidemic, while 653 (75.58%) worked outside Hubei Province, of which most of them worked in Hunan Province, the neighbour to the south of Hubei Province. The majority of nurses (61.46%) working in infection division (isolation ward), infection division (general ward), intensive care unit (ICU) or emergency room were relative with high stress (Yuwanich et al., 2015). The details of the demographic characteristics of respondents were shown in Table 1.

**TABLE 1:** Demographic characteristics of respondents (*N* = 864)

Characteristic	*N*	%
Gender		
Male	69	7.99
Female	795	92.01
Age (years old)		
18–24	85	9.84
25–39	672	77.78
40–59	107	12.38
Departments		
Infection division (isolation ward)	163	18.87
Infection division (general wards)	169	19.56
ICU or emergency room	199	23.03
Other clinical departments	310	35.88
Medical technology departments	23	2.66
Workplace		
In Hubei	211	24.42
Outside Hubei	653	75.58
Hospitals		
Designated hospitals of COVID−19 patients	597	69.10
Non‐designated hospitals of COVID−19 patients	267	39.10

### 3.2 Late‐onset PTSD symptoms

The average score of the IES‐R checklist was 27.0417 (full mark 88 points). 762 of 864 respondents (88.19%) scored more than 9 points, which can be classified as mild late‐onset PTSD symptoms. 403 of 864 respondents (46.64%) scored more than 26 points (a moderate level of late‐onset PTSD symptoms). 117 (13.54%) respondents scored more than 44 points, which can be identified as severe late‐onset PTSD symptoms. Among the 20 items of the IES‐R checklist, item 1 in the intrusion dimension got the highest mean score (2.22 points), which meant that any reminder brought back feelings about COVID‐19. The detail scores of every item were shown in Table 2a.

**TABLE 2a:** Impact of event scale‐revised items of the participants (*N* = 864)

Items	Range	Mean	*SD*
**Intrusion**	0–32	11.3993	5.5813
1. Any reminder brought back feelings about it	0–4	2.22	0.665
2. I had trouble staying asleep	0–4	1.65	1.031
3. Other things kept making me think about it	0–4	1.64	0.924
6. I thought about it when I didn't mean to	0–4	1.32	0.931
9. Pictures about it popped into my mind	0–4	1.49	0.946
14. I found myself acting or feeling like I was back at that time	0–4	1.02	0.875
16. I had waves of strong feelings about it	0–4	1.16	0.917
20. I had dreams about it	0–4	0.90	0.878
**Avoidance**	0–32	8.8715	5.91164
5. I avoided letting myself get upset when I thought about it or was reminded of it	0–4	1.45	1.012
7. I felt as if it hadn't happened or wasn't real	0–4	1.09	0.938
8. I stayed away from reminders about it	0–4	1.02	0.933
11. I tried not to think about it	0–4	1.05	0.923
12. I was aware that I still had a lot of feelings about it, but I didn't deal with them	0–4	1.22	0.909
13. My feelings about it were kind of numb	0–4	1.08	0.885
17. I tried to remove it from my memory	0–4	0.96	0.939
22. I tried not to talk about it	0–4	1.01	0.935
**Hyperarousal**	0–24	6.7708	4.57823
4. I felt irritable and angry	0–4	1.39	0.964
10. I was jumpy and easily startled	0–4	1.08	0.956
15. I had trouble concentrating	0–4	1.21	0.961
18. Reminders of it caused me to have physical reactions, such as sweating,	0–4	1.12	0.891
19. Trouble breathing, nausea, or a pounding heart	0–4	0.74	0.838
21. I felt watchful and on guard	0–4	1.22	1.017
Total	0–88	27.0417	14.88739

The average score of avoidance, intrusion, hyperarousal and total score of male nurses in the IES‐R checklist were all higher than females. This result demonstrated that male nurses were more probably to have late‐onset PTSD symptoms than females (Table 2b).

**TABLE 2b:** The comparisons of late‐onset PTSD symptoms between male and female nurses

IES‐R	Nurses (Means ± *SD*)		t	*p*
	Male (*N* = 69)	Female (*N* = 795)		
Avoidance	10.77 ± 8.71	8.71 ± 5.64	2.151	.032*
Intrusion	12.78 ± 6.18	11.28 ± 5.51	2.082	.038*
Hyperarousal	7.87 ± 5.18	6.68 ± 4.51	2.463	.016*
Total scores	31.42 ± 17.26	26.66 ± 14.61	2.222	.029*

* *p* < .05.

Compared with nurses who had worked outside Hubei Province during the epidemic, the nurses who had worked in Hubei Province got fewer late‐onset PTSD symptoms. The average score of nurses who worked outside Hubei Province during the pandemic was 27.65 (*SD* = 14.96), while the score of nurses who worked in Hubei Province during the pandemic was 25.16 (*SD* = 14.54). The difference between these two groups was statistically significant to avoidance and hyperarousal dimensions. However, there was also no statistical difference in the intrusion dimension between these two groups (Table 2c).

**TABLE 2c:** The comparisons of late‐onset PTSD between nurses who had been in/to Hubei or not during the COVID‐19 epidemic

IES‐R	Nurses who (Means ± *SD*)		t	*p*
	Has been to Hubei during pandemic (*N* = 211)	Has not been to Hubei or not during pandemic (*N* = 653)		
Avoidance	8.14 ± 5.74	9.11 ± 5.75	−2.12	.034*
Intrusion	10.93 ± 5.52	11.55 ± 5.60	−1.395	.163
Hyperarousal	6.08 ± 4.42	6.99 ± 4.61	−2.527	.012*
Total scores	25.16 ± 14.54	27.65 ± 14.96	−2.12	.034*

* *p* < .05.

### 3.3 The coping strategies of nurses

The mean scores of positive and negative coping strategies were 2.01 and 1.33, respectively, which indicated the participants tended to take positive measures against the COVID‐19 epidemic. The median of item “I tried to get away from it by eating, drinking, smoking, using drugs or medicine, etc.” and “I refuse to think too much about it” was 0, revealing that most participants would not take these measures. The details of coping strategies were shown in Table 3a. We compared the coping strategies of the “nurses who worked outside Hubei Province” group with “nurses who worked in Hubei Province” group; no statistical differences were found (Table 3b).

**TABLE 3a:** Coping strategies of the participants (*N* = 864)

Coping strategies	Range	Means	*SD*	Median
**Positive**	0–3	2.01	0.544	2
I tried to make myself feel better by working, studying, etc.	0–3	1.88	0.806	2
I confided my troubles to others	0–3	1.9	0.711	2
I tried to look on the bright side of things	0–3	2.36	0.767	3
I changed something about myself	0–3	2.21	0.834	2
I didn't take it too seriously	0–3	2.04	0.82	2
I made a plan of action and followed it	0–3	2.04	0.774	2
I found new faiths to solve the problem	0–3	2.06	0.794	2
I confided my troubles to my family, friends or colleagues	0–3	1.96	0.865	2
I changed or grew as a person in a good way	0–3	1.93	0.748	2
I drew on others experiences in the similar situation	0–3	2.03	0.751	2
I tried to make myself feel better by engaging in hobbies, leisure activities, and recreation	0–3	1.91	0.875	2
I tried to keep my feelings (e.g. sadness and anger) to myself	0–3	1.88	0.864	2
**Negative**	0–3	1.33	0.609	2
I tried to get away from it for a while by resting or taking a vacation	0–3	1.72	0.835	2
I tried to get away from it by eating, drinking, smoking, using drugs or medicine, etc.	0–3	0.76	0.9	0
I was waiting for time to change the situation	0–3	1.41	0.957	1
I refuse to think too much about it	0–3	1.09	0.934	0
I relied on others to solve the problem	0–3	1.01	0.811	1
I accepted this situation because there is nothing I can do to change it	0–3	1.55	0.881	2
I had fantasies or wishes about how things might turn out	0–3	1.17	0.92	1
I went along with fate; sometimes I just have bad luck	0–3	1.93	0.867	2

**TABLE 3b:** The comparisons of coping strategies for nurses who worked in Hubei or not during the COVID‐19 epidemic

Coping strategies	Nurses who (Means ± *SD*)		t	*p*
	Worked in Hubei (*N* = 211)	Worked outside Hubei (*N* = 653)		
Positive coping strategies scores	2.07 ± 0.53	1.99 ± 0.55	1.663	.097
Negative coping strategies scores	1.34 ± 0.55	1.33 ± 0.60	0.174	.962

### 3.4 The correlation between coping strategies and late‐onset PTSD symptoms

In order to investigate the correlation between coping strategies and late‐onset PTSD symptoms, we did a regression analysis (Table 4), which displayed that if the respondents took more positive strategies like “I tried to look on the bright side of things,” “I didn't take it too seriously,” “I confided my troubles to my family, friends or colleagues” and “I tried to make myself feel better by engaging in hobbies, leisure activities, and recreation” in the daily life, the less late‐onset PTSD symptoms they would possess (t < 0 and *p* < .05). Some items in the negative coping strategies, such as “I tried to get away from it for a while by resting or taking vacation,” “I tried to get away from it by eating, drinking, smoking, using drugs or medicine, etc.,” “I refuse to think too much about it” and “I had fantasies or wishes about how things might turn out” might lead to severer late‐onset PTSD symptoms (t > 0 and *p* < .05).

**TABLE 4:** The regressions between coping strategies and late‐onset PTSD symptoms (*N* = 864)

	Independent variable	Unstandardized coefficient		Standardized coefficient	t	*p*	VIF	*R*²	Adjusted *R*²	F
		B	SE	ß						
	Constant	25.019	2.774	‐	9.019	.000**	‐	0.278	0.261	*F* (20,843) = 16.269, *p* = .000
Positive	I tried to make myself feel better by working, studying, etc.	1.253	0.609	0.075	2.059	.04*	1.533			
	I confided my troubles to others	1.451	0.717	0.078	2.022	.044*	1.745			
	I tried to look on the bright side of things	−4.063	0.901	−0.192	−4.509	.000**	2.112			
	I changed something about myself	0.102	0.869	0.005	0.118	.906	2.211			
	I didn't take it too seriously	−2.271	0.678	−0.125	−3.35	.001**	2.219			
	I made a plan of action and followed it	−0.188	0.821	−0.01	−0.229	.819	1.621			
	I found new faiths to solve the problem	1.762	0.951	0.087	1.853	.064	2.066			
	I confided my troubles to my family, friends or colleagues	−2.081	0.83	−0.108	−2.508	.012*	2.588			
	I changed or grew as a person in a good way	−1.068	1.014	−0.05	−1.054	.292	2.163			
	I drew on others experiences in the similar situation	0.552	1.014	0.026	0.545	.586	2.646			
	I tried to make myself feel better by engaging in hobbies, leisure activities, and recreation	−2.249	0.812	−0.118	−2.769	.006**	2.707			
	I tried to keep my feelings (e.g. sadness and anger) to myself	0.462	0.758	0.024	0.609	.543	2.138			
Negative	I tried to get away from it for a while by resting or taking vacation	1.463	0.658	0.081	2.221	.027*	1.867			
	I tried to get away from it by eating, drinking, smoking, using drugs or medicine, etc.	1.28	0.621	0.081	2.061	.04*	1.55			
	I was waiting for time to change the situation	−0.445	0.648	−0.027	−0.687	.492	1.821			
	I refuse to think too much about it	3.498	0.671	0.221	5.217	.000**	2.088			
	I relied on others to solve the problem	0.057	0.749	0.003	0.077	.939	1.952			
	I accepted this situation because there is nothing I can do to change it	0.88	0.625	0.054	1.407	.16	1.695			
	I had fantasies or wishes about how things might turn out	2.213	0.67	0.136	3.305	.001**	1.983			
	I went along with fate, sometimes I just have bad luck	0.956	0.656	0.054	1.457	.146	1.615			

Note

The dependent variable: IES‐R total scores; D‐W: 0.726.

Abbreviations: SE, standard error; VIF, variance inflation factor.

* *p* < .05.

** *p* < .01.

### 3.5 The association between coping strategies and social support

Table 5a showed that the mean score of the SSRS of all participants was 42.94 (*SD* = 8.79). As shown in Table 5b, the mean score of the SSRS was 43.59 (*SD* = 8.32) of the “nurses who worked in Hubei Province” group and 42.74 (*SD* = 8.93), respectively.

**TABLE 5a:** Correlation between social supports and coping strategies of the participants (*N* = 864)

	Mean	*SD*	Positive coping	Negative coping
Subjective social support	24.1065	4.876	0.317**	−0.019
Objective social support	10.9271	4.12611	0.277**	−0.038
Support utilization	7.9109	1.85142	0.340**	−0.035
Total scores	42.9444	8.79451	0.374**	−0.036

** *p* < .01

**TABLE 5b:** The comparison of social support between nurses who has been in/to Hubei or not during the COVID‐19 epidemic

IES‐R	Nurses who (Means ± *SD*)		t	*p*
	Has been in/to Hubei (*N* = 211)	Has not been in/to Hubei (*N* = 653)		
Total scores	43.59 ± 8.32	42.74 ± 8.93	1.231	.218
Objective social support	24.39 ± 4.77	24.01 ± 4.91	0.999	.318
Subjective social support	11.20 ± 4.03	10.84 ± 4.16	1.101	.271
Support utilization	8.00 ± 1.82	7.88 ± 1.86	0.761	.447

Furthermore, according to Table 5a, the three dimensions of objective social support, subjective social support and support utilization were positively correlated with the level of positive measures participants chose to take (*p* < .05), while there was no evidence showing the correlation between these items and negative coping strategies of the participants. Furthermore, Table 5b elucidated the comparisons of social support of the “nurses who worked outside Hubei Province” group and the “nurses who worked in Hubei Province” group, but no statistical difference was detected.

## 4 DISCUSSION

Overall, the general prevalence of experiencing late‐onset PTSD was 88.19% among participants fighting against COVID‐19 during the epidemic in China. Nurses who worked in Hubei Province (the kindle place of COVID‐19 outbreak) showed a lower risk of late‐onset PTSD symptoms than those who worked outside Hubei Province. We also found that positive coping strategies were correlated with less late‐onset PTSD symptoms. Besides, social supports could help these nurses to obtain positive coping strategies.

Among our enrolled participants, 88.19% can be classified as mild late‐onset PTSD symptoms, 46.64% of the respondents showed a moderate level of late‐onset PTSD symptoms and 13.54% of the respondents can be identified as severe late‐onset PTSD symptoms. By contrast, according to a study designed to identify the prevalence of delayed‐onset PTSD in a large sample of injury survivors, of whom most were survived from the motor vehicle accident, merely 10% of traumatic injury survivors was diagnosed with 12‐month PTSD in a previous study (Carty et al., 2006). Earlier this year, a study of 14,825 participants showed that 9.1% of medical care who helped to fight against COVID‐19 in China suffered from PTSD (Song et al., 2020). It would deduce that this COVID‐19 pandemic might cause long‐term effects on individual mental health.

COVID‐19, due to its infectivity, lethality and unpredictability, could cause a huge psychological shock to frontline nurses. They have to face and bear more and more mental pressure, working load, poor sleep quality etc. (Jansson et al., 2019). Our results demonstrated that male nurses were preferential to have severer late‐onset PTSD symptoms than female ones, consistent with a previous study (Song et al., 2020). This could be explained by the fact that women are more probably to express their feelings (Street & Dardis, 2018). Moreover, because there are much fewer male nurses, they always have heavier workload than females, which may cause them to be more stressed.

Unexpectedly, nurses working in Hubei Province showed less late‐onset PTSD symptoms than those who worked outside Hubei Province during the epidemic. We found that this result was consistent with a previous study, which demonstrated that medical staff working in the Hubei province who came from other provinces were at lower risk of depressive symptoms and PTSD (Song et al., 2020). In addition, according to a study about the comparison of the psychological stress in the population of Hubei Province and non‐endemic provinces in China, people including health workers in Hubei Province tended to be less anxious and their emotional state improved during a 2‐week period (Yuan et al., 2020). We assumed that the conclusion that nurses working in Hubei Province showed less late‐onset PTSD symptoms than those who worked outside Hubei Province during the epidemic might be because nurses working in Hubei Province (the kindle place of the COVID‐19 outbreak) were well prepared and provided more training and cares. Most of these nurses went to Hubei Province voluntarily, usually with adequate psychological counselling. Furthermore, they might develop a positive emotion and be less anxious after a period of residence for the reason that they know more information about the COVID‐19 than those who worked outside Hubei Province. Finally, few medical staff assisting Hubei Province were reported to be infected with the COVID‐19 while working in the Hubei Province. Thus, nurses who worked in Hubei were not that afraid of COVID‐19, and the situation of late‐onset PTSD was better than those who worked outside Hubei Province.

We found nurses tended to take positive coping strategies facing the COVID‐19 epidemic, and this might be due to that the whole society had increased nurses' confidence and motivation by encouraging and stimulating positive emotions, so that they could gain respect, pride and satisfaction in their epidemic prevention and control (Wang et al., 2020).

There was no statistical difference between nurses working in and outside Hubei Province on coping strategies. One possible explanation is that they received similar educations and developed coping strategies to cope with the stress of their daily work. Six months after the outbreak, life has returned to normal, and their coping strategies are becoming more and more alike. Looking on the bright side of things, confiding the troubles to someone close and engaging in hobbies, leisure activities and recreation can reduce late‐onset PTSD symptoms. Refusing to think too much about it and having fantasies about how things might turn out can lead to severer late‐onset PTSD symptoms. This could give us a good guide to prevent PTSD symptoms.

Also, there was no statistical difference between nurses working in and outside Hubei Province on social support. After they got their lives back on track, they got a sufficient amount of support. According to Tesfaye's study, as mentioned earlier, social support and plan‐full problem‐solving were the most preferred strategies for nurses to cope with stress. Therefore, social support was also a key factor in nurses’ coping strategies. In our study, sufficient and effective social supports were found in Chinese clinical nurses fighting against the COVID‐19 epidemic. This result should be partly ascribed to the Chinese government and health management system for its providing enough objective support to increase nurse confidence and motivation to complete the task, such as improving working and rest condition, maintaining physical and mental health, increasing salary and allowances, priority promotion of professional title, implementing industrial injury recognition, promising their children to access to the best educational resources, employment as an establishment staff, strengthening humanistic care and improving a safer practice environment (Henan, 2020). In this study, the correlation between the tendency of nurses to take negative behaviours and the degree of social support was not statistically significant. It was assumed that nurses had sufficient social support overall so that even with relatively less social support, they still tended to take positive coping strategies rather than negative coping strategies. However, we believe that we cannot rule out the opportunity that they are relevant to some extent, resulting in the unclear definition of negative coping measures in the items of the scale. In the next experiment, if we try to further explore the correlation between negative behaviours and social measures, it will be better to re‐define and re‐list negative coping measures in a more detailed and clearer way.

### 4.1 Future implications and recommendation

These findings suggest that the government, policymakers and the whole society need to pay more attention to psychological health among nurses while combating COVID‐19. The prevalence of late‐onset PTSD symptoms is very high among Chinese frontline nurses. Not only should they get the care and attention during or shortly after the epidemic, but also the long‐term also care should never be overlooked.

The COVID‐19 has been seriously endangering public health. The nurse is one of the largest numbers of medical workers to fight against the COVID‐19 pandemic. We studied that sufficient social support of clinical nurses against the COVID‐19 pandemic is related to effective coping strategies and fewer late‐onset PTSD symptoms. Government should develop interventions for assisting clinical nurses to get more social support in such situations. For example, we can give them access to hear the voice of encouragement and gratitude from the public.

In addition, our study shows that positive coping strategies were related to fewer late‐onset PTSD symptoms. If nurses develop healthy coping strategies in their daily work, it would be easy for them to reduce the stress when handling an emergency, which may lead to fewer PTSD symptoms subsequently. For this reason, interventions from health system should throw emphasis on improving their abilities to face challenges and guiding them using healthy coping strategies against stress.

Finally, adequate protection and appropriate preferential treatment are very necessary. Measurements such as improving working and rest conditions, increasing salary and allowances, priority promotion of professional title, promising their children to access to the best educational resources inspired many nurses to work hard in combat with COVID‐19. In this way, they can devote themselves to work without worries.

### 4.2 Limitations

Despite useful findings, there are some limitations in our study. First, the information on marital status, the position and educational background of the participants were not collected. However, these items may be related to the late‐onset PTSD symptoms, coping strategies and social support to some extent. Second, we found that the correlation between the tendency of nurses to take negative behaviours and the degree of social support was not statistically significant, which is unexpected. Nevertheless, we cannot find a reasonable explanation. Thus, further study to verify this result and find the reason is required.

## 5 CONCLUSIONS

Our results suggested that Chinese frontline clinical nurses fighting against the COVID‐19 epidemic had a high risk to suffer from late‐onset PTSD symptoms 6 months after the COVID‐19 outbreak. Nurses who stayed or went to Hubei Province to fight against the COVID‐19 epidemic showed less late‐onset PTSD symptoms than those who worked outside Hubei Province. However, there were no statistical differences in coping strategies and social support between these two groups above. Chinese frontline nurses tended to take positive coping strategies rather than negative coping strategies, and positive coping strategies were related to less late‐onset PTSD symptoms. The social support Chinese nurses obtained was sufficient. We should pay more attention to the mental health problems of these nurses and give timely and sufficient help to them.

## CONFLICT OF INTEREST

None declared.

## AUTHOR CONTRIBUTIONS

All authors have agreed to the final version.

## Supporting information

App S1

## Data Availability

The data that support the findings of this study are available on request from the corresponding author. The data are not publicly available due to privacy or ethical restrictions.
