# Supplementary material for: Late‐onset PTSD and coping strategies for frontline nurses during the COVID‐19 epidemic in China
Source: Nurs Open. 2021 Aug 15;8(6):3055–64. doi: 10.1002/nop2.1018 (PMC8441903; doi:10.1002/nop2.1018)
Supplement: Supplementary file 1 — App S1 [file NOP2-8-3055-s001.docx]

**Questionnaire**

**Part 1 Basic Information**

1. Your gender
2. Male
3. Female
4. Your age
5. 0-18
6. 18-24
7. 25-39
8. 40-59
9. 60 and older
10. Your department in hospital
11. Infection Division (Isolation Ward)
12. Infection Division (General Ward)
13. ICU or emergency room
14. Other clinical departments
15. Medical technology departments
16. Is your hospital the designed hospital for COVID-19 patients or not?
17. Yes
18. No
19. Were you working in Hubei Province to fight against the COVID-19 epidemic or not?
20. Yes
21. No

**Part 2**

**Please answer the following questions based on your current status:** **(mark “√” in the appropriate box)**

| Note: “it” refers to those things which make you feel stressful during pandemic | not at all | occasionally | sometimes | often | always |
| --- | --- | --- | --- | --- | --- |
| 1. Any reminder brought back feelings about it |  |  |  |  |  |
| 2. I had trouble staying asleep |  |  |  |  |  |
| 3. Other things kept making me think about it |  |  |  |  |  |
| 4. I felt irritable and angry |  |  |  |  |  |
| 5. I avoided letting myself get upset when I thought about it or was reminded of it |  |  |  |  |  |
| 6. I thought about it when I didn’t mean to |  |  |  |  |  |
| 7. I felt as if it had not happened or was not real |  |  |  |  |  |
| 8. I stayed away from reminders about it |  |  |  |  |  |
| 9. Pictures about it popped into my mind |  |  |  |  |  |
| 10. I was jumpy and easily startled |  |  |  |  |  |
| 11. I tried not to think about it |  |  |  |  |  |
| 12. I was aware that I still had a lot of feelings about it, but I didn’t deal with them |  |  |  |  |  |
| 13. My feelings about it were kind of numb |  |  |  |  |  |
| 14. I found myself acting or feeling like I was back at that time |  |  |  |  |  |
| 15. I had trouble falling asleep |  |  |  |  |  |
| 16. I had waves of strong feelings about it |  |  |  |  |  |
| 17. I tried to remove it from my memory |  |  |  |  |  |
| 18. I had trouble concentrating |  |  |  |  |  |
| 19. Reminders of it caused me to have physical reactions, such as sweating, trouble breathing, nausea, or a pounding heart |  |  |  |  |  |
| 20. I had dreams about it |  |  |  |  |  |
| 21. I felt watchful and on guard |  |  |  |  |  |
| 22. I tried not to talk about it |  |  |  |  |  |

**Part 3**

**Please answer the following questions based on your current status:**

1. How many close friends do you have who can get support and help? (please select one only)

A. None

B. 1-2

C. 3-5

D. 6 or more

2. In the past year you: (please select one only)

A. Stay away from family members and live alone.

B. often change residence, mostly living with strangers.

C. Live with classmates, colleagues or friends.

D. Live with family.

3. You and your neighbor: (please select one only)

A. Never care about each other.

B. May be a little concerned when encountering difficulties.

C. Some neighbors care about you very much.

D. Most neighbors care about you very much.

4. You and colleagues: (please select one only)

A. Never care about each other, just nod their heads.

B. May be a little concerned when encountering difficulties.

C. Some colleagues care about you very much.

D. Most colleagues care about you.

5. Support and care received from family members (mark “√” in the appropriate box)

|  | None | Few | Average | Abundant | Assuming that the total family support score is 10, please fill in the percentage of A.B.C.D.E. |
| --- | --- | --- | --- | --- | --- |
| A. Couple (lovers) |  |  |  |  |  |
| B. Parents |  |  |  |  |  |
| C. Children |  |  |  |  |  |
| D. Brothers and sisters |  |  |  |  |  |
| E. Other members (such as sister-in-law) |  |  |  |  |  |

6. In the past, when you encountered an emergency, the sources of financial support and help to solve practical problems were:

(1) None.

(2) The following sources: (multiple options can be selected)

A. Spouse

B. Other family members

C. Friends

D. Relatives

E. Colleague

F. Work unit

G. Official or semi-official organizations such as Party, League, Trade Union, etc.

H. Religious, social organizations and other non-official organizations

I. Others (please list)

7. In the past, when you encountered an emergency situation, the sources of comfort and concern you received were:

(1) None.

(2) The following sources: (multiple options can be selected)

A. Spouse

B. Other family members

C. Friends

D. Relatives

E. Colleague

F. Work unit

G. Official or semi-official organizations such as Party, League, Trade Union, etc.

H. Religious, social organizations and other non-official organizations

I. Other (please list)

8. How to talk when you encounter troubles: (please select one only)

A. Never tell anyone.

B. Only report to one or two individuals who are extremely closely related.

C. If a friend asks, you will tell it.

D. Take the initiative to tell your troubles to gain support and understanding,

9. How to ask for help when you encounter troubles: (please select one only)

A. Rely on yourself and don't accept help from others.

B. Rarely ask for help from others.

C. Sometimes ask for help from others.

D. Frequently ask for help from family members, relatives, friends, and organizations when in difficulties.

10. For groups activities (such as party organizations, religious organizations, trade unions, student unions, etc.), you: (please select one only)

A. Never participate.

B. Participate occasionally.

C. Participate often.

D. Take the initiative to participate and be active.

**Part 4**

**Please answer the following questions based on your attitude or strategies adopted in the face of the COVID-19.** **(mark “√” in the appropriate box)**

|  | not at all | occasionally | sometimes | often | always |
| --- | --- | --- | --- | --- | --- |
| 1. I tried to make myself feel better by working, studying, etc. |  |  |  |  |  |
| 2. I asked advice from a relative, friend or classmate |  |  |  |  |  |
| 3. I tried to look on the bright side of things |  |  |  |  |  |
| 4. I changed something about myself |  |  |  |  |  |
| 5. I didn’t take it too seriously |  |  |  |  |  |
| 6. I made a plan of action and followed it |  |  |  |  |  |
| 7. I found new faiths to solve the problem |  |  |  |  |  |
| 8. I confided my troubles to my family, friends or colleagues |  |  |  |  |  |
| 9. I changed or grew as a person in a good way |  |  |  |  |  |
| 10. I drew on others experiences in the similar situation |  |  |  |  |  |
| 11. I tried to make myself feel better by engaging in hobbies, leisure activities, and recreation |  |  |  |  |  |
| 12. I tried to keep my feelings (e.g., sadness and anger) to myself |  |  |  |  |  |
| 13. I tried to get away from it for a while by resting or taking vacation |  |  |  |  |  |
| 14. I tried to get away from it by eating, drinking, smoking, using drugs or medicine, etc. |  |  |  |  |  |
| 15. I was waiting for time to change the situation |  |  |  |  |  |
| 16. I refuse to think too much about it |  |  |  |  |  |
| 17. I relied on others to solve the problem |  |  |  |  |  |
| 18. I accepted this situation because there is nothing I can do to change it |  |  |  |  |  |
| 19. I had fantasies or wishes about how things might turn out |  |  |  |  |  |
| 20. I went along with fate, sometimes I just have bad luck |  |  |  |  |  |
